# Generation of effective and specific human TCRs against tumor/testis antigen NY-ESO-1 in mice with humanized T cell recognition system

**DOI:** 10.3389/fimmu.2024.1524629

**Published:** 2024-12-24

**Authors:** Xiaojing Tina Chen, Matthias Leisegang, Ioannis Gavvovidis, Seth M. Pollack, Felix K. M. Lorenz, Ton N. Schumacher, Oliver Daumke, Thomas Blankenstein

**Affiliations:** ^1^ Molecular Immunology and Gene Therapy, Max Delbrück Center for Molecular Medicine in the Helmholtz Association (MDC), Berlin, Germany; ^2^ Institute of Immunology, Charité Universitätsmedizin, Berlin, Germany; ^3^ David and Etta Jonas Center for Cellular Therapy, the University of Chicago, Chicago, IL, United States; ^4^ Institute of Immunology, Charité - Universitätsmedizin Berlin, corporate member of Freie Universität Berlin and Humboldt-Universität zu Berlin, Berlin, Germany; ^5^ Fred Hutchinson Cancer Research Center, University of Washington, Seattle, WA, United States; ^6^ Max Delbrück Center for Molecular Medicine in the Helmholtz Association (MDC), Berlin, Germany; ^7^ Division of Molecular Oncology & Immunology, Oncode Institute, The Netherlands Cancer Institute, Amsterdam, Netherlands; ^8^ Department of Hematology, Leiden University Medical Center (LUMC), Leiden, Netherlands; ^9^ Structural Biology, Max Delbrück Center for Molecular Medicine in the Helmholtz Association, Berlin, Germany

**Keywords:** adoptive T cell therapy, TCR engineering, humanized mice, NY-ESO-1, HLA-A*0201

## Abstract

Generation of high avidity T cell receptors (TCRs) reactive to tumor-associated antigens (TAA) is impaired by tolerance mechanisms, which is an obstacle to effective T cell therapies for cancer treatment. NY-ESO-1, a human cancer-testis antigen, represents an attractive target for such therapies due to its broad expression in different cancer types and the restricted expression in normal tissues. Utilizing transgenic mice with a diverse human TCR repertoire, we isolated effective TCRs against NY-ESO-1_157-165_ restricted to HLA-A*02:01. We compared the functions of the murine-derived TCR with human-derived TCRs and an affinity matured TCR, using *in vitro* co-culture and *in vivo* adoptive T cell transfer in tumor-bearing mice. Alanine scan, x-scan, LCL assay were employed to address the cross-reactivity of the NY-ESO-1_157-165_ specific TCRs. We also used human tissue cDNA library and human primary cells to assess the safety of adoptive T cell therapies targeting NY-ESO-1 antigen in the clinic. One of the murine-derived human TCRs, TCR-ESO, exhibited higher functional avidity compared to human-derived NY-ESO-1_157-165_ specific TCRs. TCR-ESO appeared to have similar efficiency in antigen recognition as an *in vitro* affinity-matured TCR, TCR 1G4-α95LY, which was applied in clinical trials. TCR-ESO showed little cross-reactivity, in contrast to TCR 1G4-α95LY. Our data indicate that highly effective TCRs against NY-ESO-1 are likely deleted in humans due to tolerance mechanisms, and that the TCR gene loci transgenic mice represent a reliable source to isolate effective and highly-specific TCRs for adoptive T cell therapies.

## Introduction

Upon engaging the antigens with their T cell receptor (TCRs), CD8 T cells can recognize their cellular targets and exert cytotoxic functions to eliminate the targets. Many immunotherapies exploit such features of CD8 T cells for cancer treatment, e.g. it was suggested that the number of infiltrated CD8 T cells correlates positively with clinical outcomes in immune checkpoint blockade therapy (1). Another example is adoptive T cell therapy (ATT). By introducing tumor antigen-specific TCRs or chimeric antigen receptors (CARs) into patients’ T cells and re-infusing them into the patient, redirected T cells can recognize and eliminate cancer cells. Several ATT trials have shown encouraging clinical responses with low or affordable toxicities (2–5). Among ATTs, the TCR-T therapies showed promising effects in solid tumors (6).

One facet that determines the success of ATTs is the selection of tumor antigens. Ideally, the expression of the target antigens should be restricted to cancer cells (tumor-specific antigens, e.g. neoantigens), which excludes potential on-target off-tumor toxicity (7). Yet, these kind of antigens require highly individualized treatment approaches. They must be expressed in sufficient amounts and be endogenously processed and presented by the patient’s HLA-I molecules (human leukocyte antigen-I molecules) as 8-12mer peptides, making them exquisite but rare targets. Targeting tumor associated antigens (TAAs) represents an alternative, if the antigen is not expressed in normal tissues or only in limited amounts. Cancer-testis (CT) antigens are widely expressed in different cancer types (8). Their expression in normal tissues is limited to testis and sometimes also to placenta, which are known immune-privileged sites (9, 10), making these group of antigens attractive targets for ATT.

NY-ESO-1 is a CT antigen that was detected at various frequencies in solid tumors (11), e.g. it is expressed in 25-50% of melanomas or up to 80% of synovial sarcomas (12, 13). An HLA-A0201 epitope of NY-ESO-1, NY-ESO-1_157-165_, has been previously described (14). Clinical trials of ACT against NY-ESO-1_157-165_ have been conducted in multiple myeloma, metastatic melanoma and synovial sarcoma patients. In these trials, more than half of the patients from different cancer types responded to the therapy with no toxicities observed (3, 15), suggesting NY-ESO-1 as a safe target for ATT.

Another critical factor that contributes to successful ATT is the generation of tumor antigen-specific TCRs. The TCRs should endow T cells with high potency in anti-tumor activity and have no cross-reactivity in order to prevent off-target toxicity. TCRs can be isolated from humans, for example from tumor-infiltrating lymphocytes (TILs) (16). Due to immune tolerance, human-derived TCRs against TAAs often have low avidity and require *in vitro* maturation (17, 18). The *in vitro* selection steps could introduce cross-reactivity to the TCR variants, which was exemplified by a clinical report utilizing an affinity-enhanced MAGE-A3-specific TCR. The administration of the TCR-expressing T cells caused lethal cardiac toxicity due to TCR recognition of a peptide derived from the striated muscle-specific protein titin (18, 19). Allogeneic T cell priming is another approach, however, the majority of allogeneic TCRs are primarily reactive towards the HLA molecules rather than the peptide (20). HLA-transgenic mice have also been employed as non-tolerant hosts to isolate high avidity T cells against human tumor antigens (21, 22). The drawback is the potential recognition of murine TCRs by the patient immune system. Furthermore, potential incompatibility between murine TCRs and human MHC molecules might lead to sub-optimal TCR potency (23).

Previously, we reported the generation of transgenic mice that contain a complete humanized T cell recognition system (23, 24). These mice contain human TCRα/β gene loci and are defective for mouse TCRα/β expression. In these mice, human MHC I or II molecules were introduced and the murine MHC molecules were knocked out. Thus, the problem of species incompatibility between human and mouse MHC-TCRs was overcome. One of the strains, called AB*ab*-A2 (previously AB*ab*DII) mice, expresses HLA-A*0201 molecules. TCRs isolated from AB*ab*-A2 mice against a CT antigen, MAGE-A1 were proven to be highly efficient in MAGE-A1^+^HLA-A*02:01^+^ tumor cell recognition, both *in vitro* and *in vivo (* 25).

NY-ESO-1 was detected in human medullary thymic epithelial cells (26), indicating that thymic selection might lead to the deletion of high avidity T cells in humans. NY-ESO-1 has no known homologs in mice, meaning that mice should not be tolerant to NY-ESO-1. AB*ab*-A2 mice may be a viable source for isolating highly potent NY-ESO-1_157-165_ reactive TCRs. Here, we identified an AB*ab*-A2-derived NY-ESO-1_157-165_ specific TCR that conferred T cells with high avidity in target cell recognition, both *in vitro* and *in vivo*. The mouse-derived TCR showed higher potency than TCRs isolated from humans, and had comparable sensitivity and functionality with the clinical relevant TCR 1G4-α95LY. TCR 1G4-α95LY is an affinity matured variant of TCR 1G4 which was derived from the PBMCs of a melanoma patient and tested in clinical trials (2, 15). In TCR 1G4-α95LY, the threonine at P95 was replaced by leucine and serine at P96 by tyrosine in the TCRα chain of TCR 1G4 (17, 27). The murine-derived TCR appeared to be specific, with few cross-reactivities to other peptides presented by HLA-A*0201 and other HLA-I molecules. We also showed that the TCR 1G4-α95LY recognition motif was more promiscuous in comparison to the murine-derived TCR, and our safety screening for TCR 1G4-α95LY indicated a certain degree of cross-reactivity.

## Materials and methods

### Peptides

NY-ESO-1_157-165(165CtoV)_, alanine scan and x-scan peptides were synthesized (Genscript) and dissolved in 20% DMSO.

### Cell lines

The human myeloma cell line U266 (25), the human melanoma cell lines 624.28Mel, 624.38Mel, Mel285, SK.Mel29, SK.Mel29.NY were kept in RPMI 1640 supplemented with 10% FCS (20). The human myxoid liposarcoma cell lines 1765 (28) and FUJI were retrovirally transduced with HLA-A0201 and were cultured in RPMI supplemented with 10% FCS. The TCR deficient Jurkat cell line was kept in RPMI supplemented with 10% FCS. T2 cells were kept in RPMI supplemented with 10% FCS. The viral packaging cells 293GP-GLV and PlatE cells were cultured in DMEM supplemented with 10% FCS. Epstein-Barr virus–transformed lymphoblastoid B cell lines (B-LCLs) were cultured in RPMI 1640 supplemented with 10% FCS, 50 μM 2-mercaptoethanol, 1 mM sodium pyruvate and 1x nonessential amino acids. The murine MC703.gCm line was cultured in RPMI supplemented with 10% FCS. Routine mycoplasma testing was performed.

### Mice

AB*ab*-A2 mice express a human TCR repertoire and a mouse/human chimeric molecule HHD. HHDxRag^–/–^ mice were generated in house by crossing the Rag^-/-^ strain with the HHD strain (25). Mice were taken at age 6–16 weeks for experiments. All mouse lines were bred and kept under specific pathogen–free conditions (SPF). All animal experiments were performed according to institutional and national guidelines and regulations (Landesamt für Gesundheit und Soziales, Berlin).

### Immunization

For peptide immunization, 100 µg of synthetic NY-ESO-1_157-165_ peptide (SLLMWITQV, Genescript) was mixed with 50 µg CpG1826 (MOLBIOL) in 100 µl PBS and an emulsion was prepared by mixing the peptide-CpG with 100 µl incomplete Freund’s adjuvant. The mixture was injected into the ABabA2 mice subcutaneously. For full antigen immunization, the Helios Gene Gun system (Bio-Rad) was used. In brief, pcDNA3.1 encoding codon optimized full length NY-ESO-1 gene/GM-CSF were attached onto 0.6 µm gold Microcarriers, loaded to the Tefzel tubing and processed the tubing into cartridges using the Tubing Prep Station from Bio-Rad. The DNA-Microcarriers was delivered to the mice on skin of the lowered abdomen using pressurized gene gun. Immunizations were carried out with an interval of 4 weeks. Blood was taken 7 days post immunization, spleen was isolated at day 10 after boost. Response was measured either by NY-ESO-1_157-165_/A2 tetramer staining or intracellular staining of murine IFNγ production (ICS, BD Biosciences). For ICS, T cells from blood or spleen was stimulated by 10^-6^ M NY-ESO-1_157-165_ peptide, 1:1000 GolgiPlug (BD Biosciences) was added to the co-culture 2 hours after incubation, and the co-culture was carried out for another 12 hours. The cells were then fixed and staining for surface markers and IFNγ.

### Retroviral transduction of NY-ESO-1 TCRs

The human constant regions of the TCRs used in this study were replaced by murine constant regions. TCR gene cassette were codon-optimized for mammalian expression and synthesized by GeneArt. The TCRs were constructed into MP71-PRE vector as described previously (25). The MP71-TCRs were transfected into the viral packaging lines 293GP-GLV (amphotropic) or Plat-E (ecotropic) using Lipofectamine 2000 reagent (Thermo Fisher Scientific), and the supernatant containing the viral particles were harvested 24 and 48h after transfection. The human PBMCs were activated for 48h with anti-human CD3/CD28 antibodies and 200 U/ml human IL2 prior to viral infection. For mouse T cell infection, murine splenocytes were isolated and activated with anti-murine CD3/CD28 beads (Thermo Fisher Scientific) and 100 U/ml murine IL2 for 24h prior to viral infection. Two transductions were done for each experiment: the first transduction was carried out by spinoculation of the viral supernatant with the cells, at 800xg for 90min and 32°C; the next day, the 48h viral supernatant was attached to Retronectin (Takara) coated plates by centrifugation at 3200xg for 90min at 4°C, and the cells were added onto the viral coated plates and spin for an additional 30 min at 32°C. The cells were kept at 200 U/ml IL2 for 10 days and at 20 U/ml IL2 for 3 additional days before use.

### Co-culture experiments

Co-culture experiments with human cells were performed by incubating 5x10^4^ transduced TCR^+^CD8^+^ T cells with 5x10^4^ target cells unless if specified. For co-culture with mouse cells, 1x10^5^ transduced CD8 T cells were co-cultured with 1x10^5^ target cells. IFNγ production was measured 16-18h after co-culture from the supernatant by ELISA (BD Biosciences).

### Flow cytometry

Antibodies were purchased from BioLegend unless otherwise indicated: anti-mCD8 (clone 53-6.7), anti-mCD3 (145-2C11, 1:200), anti–mIFN-γ–BV421 (XMG1.2), anti–mTCRβ chain (H57-597) anti-hCD3 (SK7), anti-hCD8 (SK1), NY-ESO-1_157-165CtoV_/A2Kb which contains murine H-2Kb α3 domain in replacement of human α3 and NY-ESO-1_157-165CtoV_/A2 tetramer were generated in-house.

### ATT of NY-ESO-1 specific T cells

MC703.NY cancer cells were inoculated subcutaneously in the flank of HHDxRag^-/-^ mice and were allowed to grow for 3 weeks before T cell transfer. After 3 weeks, 5x10^5^ TCR transduced HHD CD8 T cells were transferred by intravenous injection into the mice. The size of the tumor was monitored every 2 days. The blood was taken on day 10, 20 and 30 to analyze the number of adoptively transferred T cells. Each group of the ATT experiment included 4-5 mice to reach statistic significance. The mice were euthanized if the tumor size reached 1500 mm^3^ or when reached the end point of the experiment. The conductor of the experiment was aware of the group allocation.

### Structural biology

Figures were prepared with the PyMOL Molecular Graphics System, Version 2.5.5, Schrödinger. TCRα residues 95 and 96 were exchanged in Coot (29) to a preferred conformer.

### FACS

FACSAria II was used for sorting of cells, and FacsCanto II or LSRFortessa was used to analyze cells by flow cytometry (BD).

### Statistics

All the statistics in this study used 2-tailed t test using GraphPad Prism 7. P values of 0.05 or less were considered significant.

For animal experiments sample size was estimated based on the primary endpoint of tumor size 14 days after T cell injection. Sample size for animal experiments was estimated to detect a standardized effect size of 1.2 with a power of 80% and a dropout rate of 10%, leading to 8-9 animals per group. Mean tumor volumes between groups were compared using one way ANOVA.

## Results

### Robust CD8 T cell response against NY-ESO-1_157-165_ can be elicited in ABab-A2 mice

There is no homologue of NY-ESO-1 in mice, and NY-ESO-1_157-165_ shares only 6 out of 9 amino acids with the most closely related murine peptide sequence (XK-related protein 8) (**Supplementary Figure 1A**). Mice are, therefore, unlikely tolerant towards NY-ESO-1_157-165_ and represent a good source to isolate optimal-avidity CD8 T cells against the antigen. Optimal-avidity CD8 T cells are defined here as those with TCRs that are restricted to self-MHC molecules and recognize the epitope as foreign, e.g. similar to pathogen-specific CD8 T cells.

**Figure 1:**
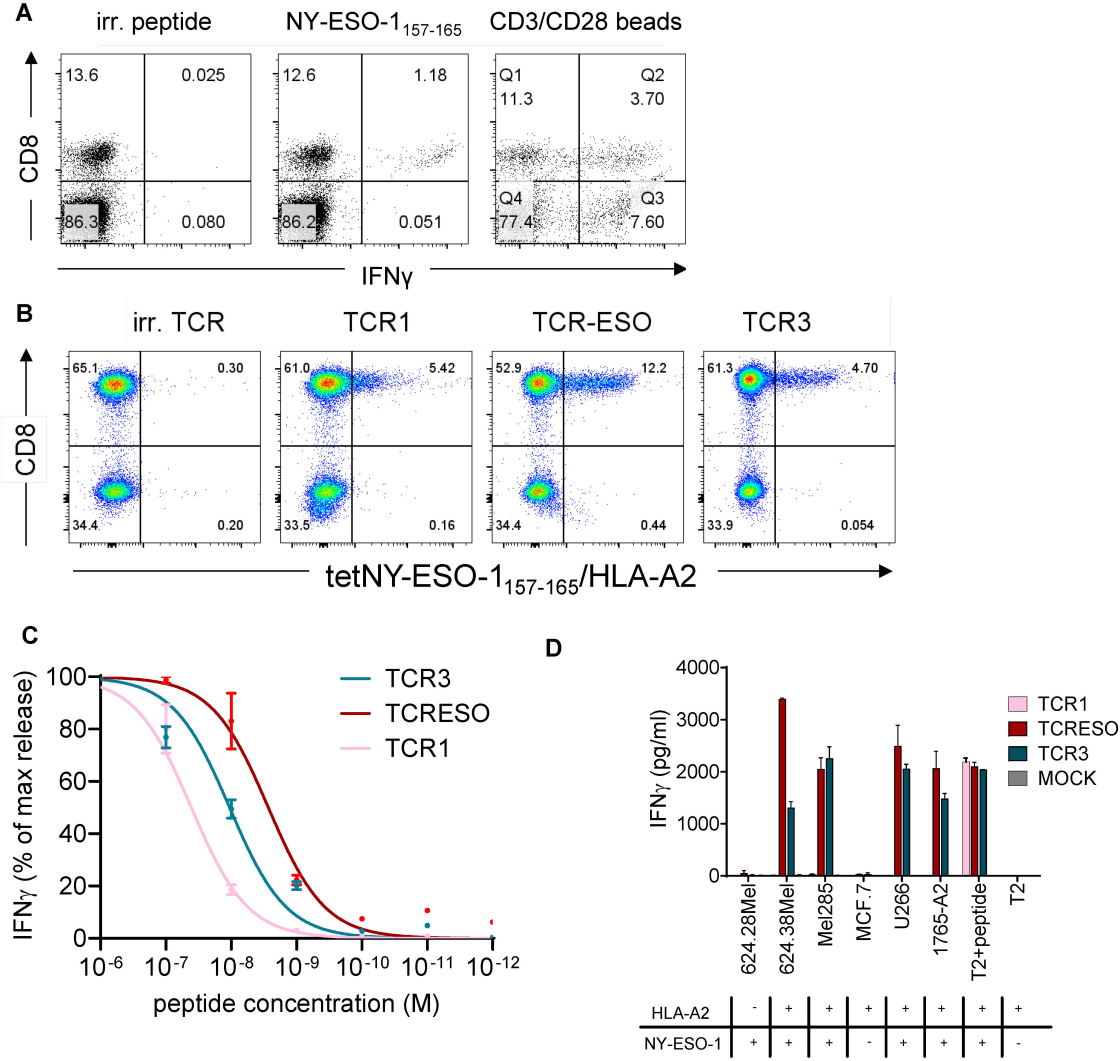
**(A)** IFNγ intracellular staining of blood cells of NY-ESO-1 immunized AB*ab*-A2 mice. AB*ab*-A2 mice were gene gun immunized and boosted with NY-ESO-1 full length cDNA. Blood was drawn 7 days after the last boost and cells re-stimulated for 14 hours with MAGE-A1_278_ (irrelevant peptide), NY-ESO-1_157-165(9CtoA)_ peptide or anti-mouse CD3/CD28 beads and stained intracellularly for IFNγ production. One representative experiment of 21 immunized mice is shown. **(B)** Re-expression of TCR1, TCR-ESO and TCR3 in human PBMCs. Human PBMCs were transduced with NY-ESO-1_157-165_ TCRs encoding retrovirus and stained with NY-ESO-1_157-165_/HLA-A2 tetramer. **(C)** Peptide titration of TCR1, TCR-ESO and TCR3 transduced huPBMCs. Different concentration of NY-ESO-1_157-165_ peptide were loaded onto T2 cells as targets for TCR transduced huPBMCs and cultured overnight. IFNγ levels were then measured by human IFNγ ELISA. One-site specific binding curves were calculated on the normalized IFNγ secretion level. Combined data from 3 independent experiments. **(D)** Tumor cell recognition by TCR1, TCR-ESO and TCR3 transduced human PBMCs. Each 5x10^4^ effector and target cells (1:1) were co-cultured in duplicates for 16-18h. IFNγ levels were measured by ELISA. The experiment was repeated once and both yielded similar results. See also **Supplementary Figure 1**.

In this study, we utilized AB*ab*-A2 mice, which contain the complete human TCR α and β gene loci and the human MHC-I gene HLA-A*02:01 in their genome. The human MHC I gene is expressed as chimeric molecule with the α3 domain derived from mouse H-2D^b^ to allow CD8 receptor binding and is fused to human β2m. Additionally, the murine TCR α and β constant region genes and murine MHC-I (β2m and H-2D^b^) have been knocked out. Hence, the mice have a humanized T cell recognition system. Different immunization regimens were administered to the mice, including peptide immunization-boost, full NY-ESO-1 cDNA immunization-boost and a combinatory immunization of full antigen immunization and peptide boost. To note, in order to prevent oxidation of the NY-ESO-1_157-165_ peptide due to possible disulfide bond formation of the cysteine in position 9 of the epitope, we used an analogue, NY-ESO-1_157-165(C165toV)_, for peptide immunization (30). Specific CD8 T cell responses were detected 7 days after the final boost in all three regimens, the percentages of specific CD8 T cells ranged from ~2% to more than 20% of all CD3 T cells (**Figure 1A**; **Supplementary Figure 1B**). We selected two mice that responded robustly to the combinatory immunization for TCR identification.

### TCR-ESO shows superior functionality among all isolated NY-ESO-1_157-165_ TCRs from AB*ab*-A2 mice

CD8^+^ T cells from the immunized AB*ab*-A2 mice that bound an NY-ESO-1_157-165_ loaded chimeric HLA-A*02:01 tetramer (α3 domain derived from H-2K^b^) were enriched by flow cytometry (purity >99%) (**Supplementary Figure 1C**) and the paired TCRα and β sequences were identified using TCR capture assay (31) (see **Supplementary Figure 1D** for TCR sequences). To test the specificity of the identified TCRs, the paired TCRs were retrovirally transferred into Jurkat76 cells, which are devoid of endogenous TCRs (**Supplementary Figure 1E**). TCR1-, TCR-ESO- and TCR3-transduced CD8-expressing Jurkat76 cells bound to NY-ESO-1_157-165_/A2 tetramer to different extent and were subjected to further analysis.

In order to increase the expression and to prevent mispairing with endogenous TCRs, we replaced the human constant regions of the three TCRs, Cα and Cβ, with murine counterparts. All three chimeric TCRs were expressed in primary human T cells (**Figure 1B**). The TCR-transduced human T cells showed different sensitivities to stimulation by TAP-deficient T2 cells loaded with titrated amounts of NY-ESO-1_157-165_ peptide (**Figure 1C**). TCR-ESO transduced T cells detected the lowest amount of peptide compared to the other two TCRs. The three TCR-transduced human T cells also recognized NY-ESO-1^+^A2^+^ human tumor lines to different degree, with TCR-ESO-expressing T cells secreting the highest amount of IFNγ upon tumor cell recognition (**Figure 1D**). TCR1 did not recognize endogenously processed and presented NY-ESO-1 antigen, most likely due to the low avidity of the T cells transduced with this TCR. TCR-ESO was selected for further analysis because it showed improved recognition of naturally occurring NY-ESO-1-expressing cancer cells compared to TCR3.

### TCR-ESO endowed higher avidity to T cells compared to human-derived TCRs

**Figure 2:**
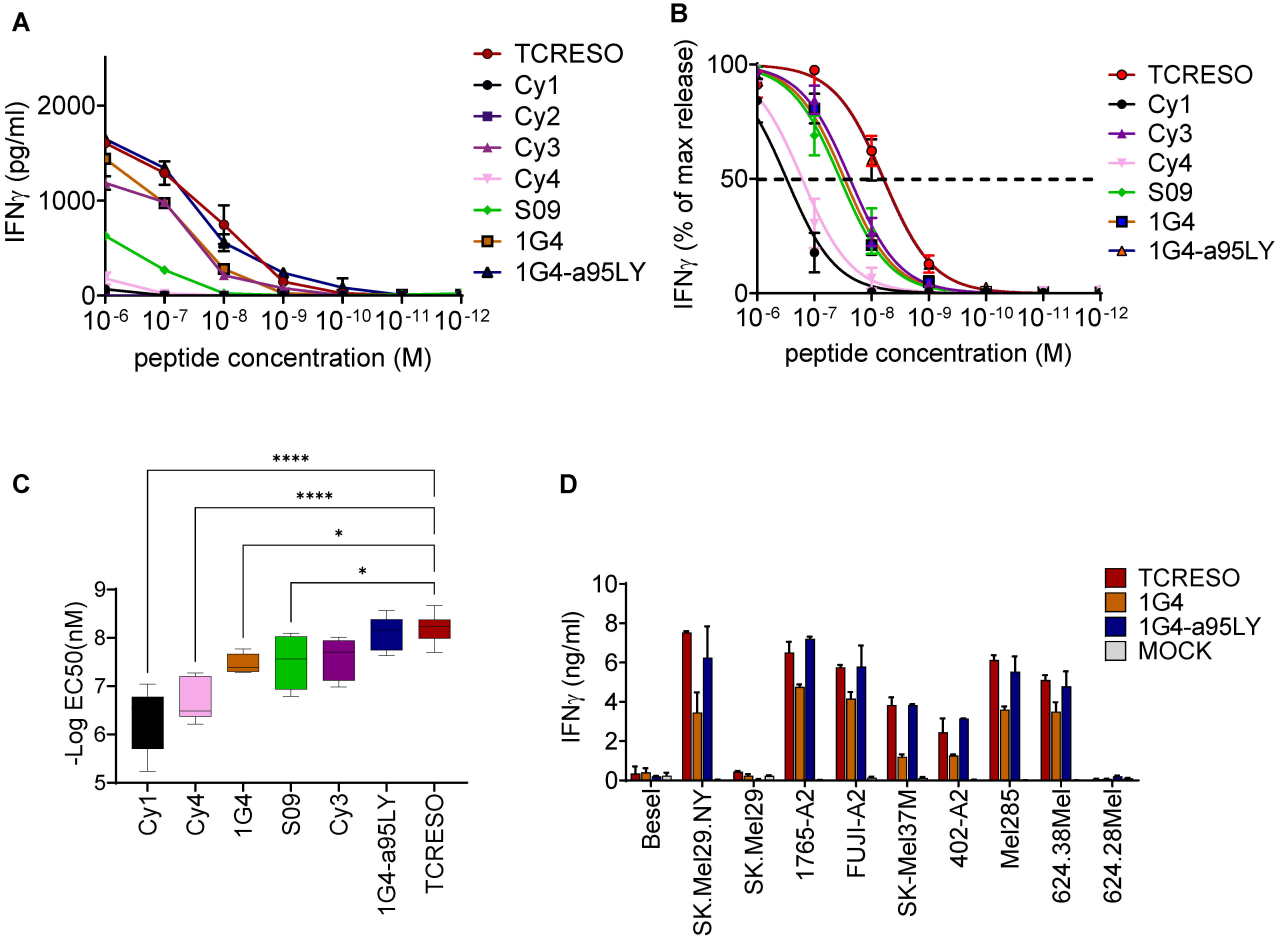
Affinity comparison of NY-ESO-1_157-165_ specific TCRs isolated from different source. **(A)** Recognition of T2 cells loaded with different amount of NY-ESO-1_157-165_ peptides by different NY-ESO-1_157-165_ specific T cells. Human PBMCs were transduced with NY-ESO-1_157-165_ TCRs from ABab-A2 mice (TCR-ESO), human donors (Cy1, Cy3, Cy4, S09 and TCR 1G4) or *in vitro* mutagenized TCR from human (TCR 1G4-α95LY). Cy2 TCR was used as negative control. 10^4^ transduced CD8 were cultured with 10^4^ T2 cells loaded with different amounts of NY-ESO-1_157-165_ wildtype peptide as indicated overnight. **(B)** IFNγ level was normalized to the highest secretion level at peptide concentration of 10^-6^ M. One-site specific binding curves were then calculated from the normalized IFNγ secretion level. Pooled data from three human donors are shown. The assay was repeated in two or three independent experiments with each donor with similar results. **(C)** Comparisons of the EC50 of the TCRs calculated from **(B)**. one way ANOVA was applied to compared the EC50s and * indicates a P value <0.05 and **** indicates a P value <0.0001. **(D)** Recognition of tumor cell lines expressing NY-ESO-1 and HLA-A*02:01. 5x10^4^ transduced enriched human CD8 T cells were cultured overnight with 5x10^4^ tumor cells. Expression of NY-ESO-1 and/or HLA-A2 is indicated. The T cell recognition was assessed by human IFNγ ELISA with the culture supernatant. The assay was repeated in two independent experiments with each human donor, three human donors were used in this experiment. See also **Supplementary Figure 2**.

NY-ESO-1 was shown to be expressed in human medullary thymic epithelial cells (26), which may lead to central tolerance. Although NY-ESO-1 specific T cells that arise in humans have been reported (14, 32, 33), one could anticipate low avidity of these T cells. We compared the avidity of TCR-ESO T cells with the avidity of human-derived TCRs, which included Cy1, Cy3, Cy4 and S09 from synovial sarcoma and myxoid/round cell liposarcoma (MRCL) patients, and 1G4 from a melanoma patient (34) (**Supplementary Figure 2A** for TCR sequences). In addition, we also included 1G4-α95LY in the comparison. TCR 1G4-α95LY is an *in vitro*-functionally enhanced mutant of the original TCR 1G4. We assessed the TCR potency in sorted primary human CD8 T cells (**Supplementary Figure 2B**). TCR-ESO and TCR 1G4-α95LY transduced CD8 T cells secreted similar amount of IFNγ, and were sensitive to stimulation by as low as 10^-9^ M NY-ESO-1_157-165_ wildtype peptide (IC50 of ~10^-8^ M) (**Figures 2A–C**). All unmodified human-derived NY-ESO-1 TCR-expressing CD8 T cells showed weaker responses towards peptide stimulation in terms of amount of IFNγ production and sensitivity to titrated amounts of peptide (**Figures 2A–C**).

We examined the capacity of ABab-A2- and of human-derived TCRs to recognize endogenously processed NY-ESO by co-culturing of TCR-transduced T cells with tumor cell lines expressing NY-ESO as well as HLA-A2. TCR-ESO and TCR 1G4-α95LY recognized the NY-ESO-1^+^A2^+^ cell lines at comparable recognition levels. TCR 1G4 CD8 T cells recognized the tumor line at lower level (**Figure 2D**).

**Figure 3:**
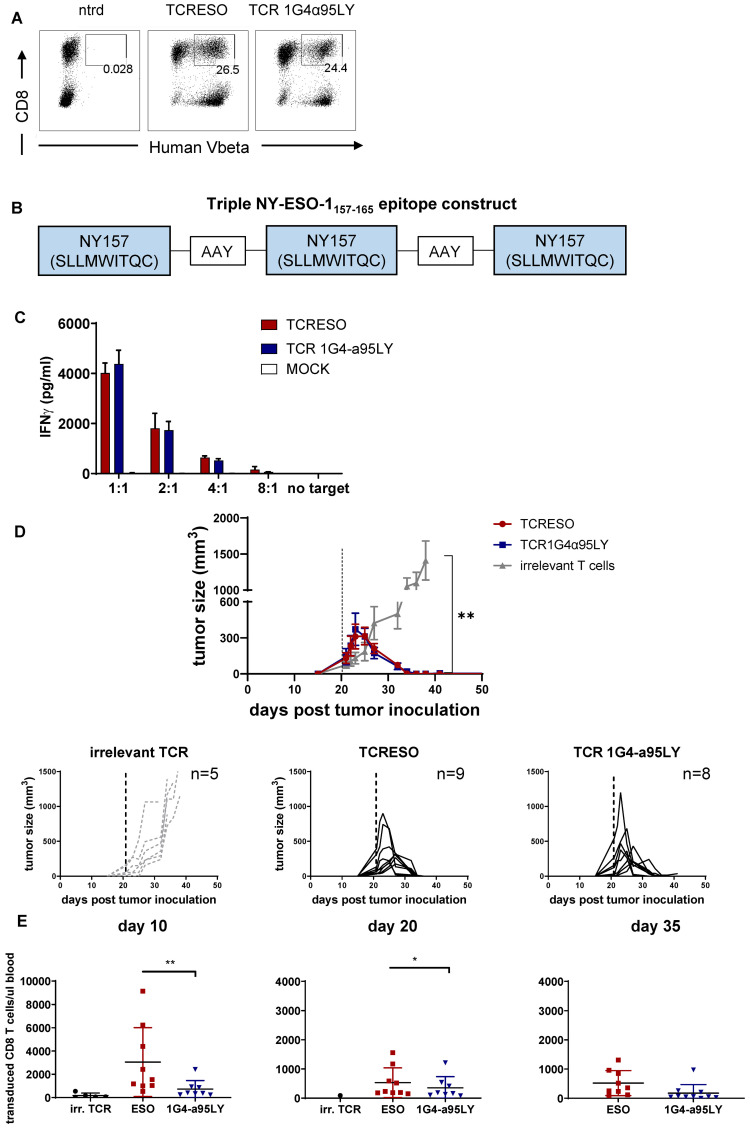
Adoptive T cell therapy of HHDxRag^-/-^ mice bearing mice with NY-ESO-1_157-165_ specific T cells. HHDxRag^-/-^ mice were inoculated with 5x10^6^ MC703.gCm tumor cells and treated with 5x10^5^ transduced HHD CD8 T cells. **(A)** Human TCRVβ FACs staining (TCR-ESO: Vβ8; TCR 1G4-α95LY: Vβ13.1) of NY-ESO-1_157-165_ TCR transduced HHD splenocytes. Gate: CD3^+^ lymphocytes. **(B)** Illustration of the organization of the NY-ESO-1_157-165_ triple epitope cassette introduced into MC703 cells. **(C)** Recognition of MC703-NY cell line by NY-ESO-1_157-165_ TCR transduced HHD splenocytes. 10^4^ transduced HHD splenocytes were cultured overnight with different numbers of MC703.NY cells. The experiment was done in duplicates. Non-transduced HHD splenocytes were used as negative control. **(D)** MC703-NY tumor growth curve in HHDxRag^-/-^ mice. The dashed line indicates the day of adoptive T cell transfer. The experiment was done in parallel for the two NY-ESO-1 TCRs and irrelevant T cells. Upper panel: average tumor size curve. Lower panels: tumor curves of individual mice treated with the three different T cells (TCR-ESO, TCR 1G4-α95LY, irrelevant TCR). **(E)** Specific CD8 T cell counts in the blood of tumor-bearing mice on day 10, 20 and 35 after T cell transfer. The experiment was repeated once (combination of both experiments, each experiment includes 4-5 mice/group).

We further compared the potency of TCR-ESO and TCR 1G4-α95LY *in vivo*. For this purpose, we transduced the two TCRs into T cells from HHD mice (**Figure 3A**). HHD mice are transgenic mice that express a chimeric HLA-A*02:01 molecule with H-2D^b^ α3 domain (35). The transduced murine T cells were co-cultured with a murine fibrosarcoma line, MC703 which was modified to express NY-ESO-1_157-165_ triple epitope (MC703-NY, **Figure 3B**) (36). TCR-ESO and TCR 1G4-α95LY expressing murine T cells secreted similar amounts of murine IFNγ upon co-culture, mirroring the findings observed in human T cells (**Figure 3C**).

5x10^6^ MC703-NY cells were injected subcutaneously into HHDxRag1^-/-^ mice and tumors were allowed to grow for more than three weeks to reach sizes of 50-400 mm^3^. 5x10^5^ TCR transduced HHD CD8 T cells were then injected i.v. into the tumor-bearing mice to exert their anti-tumor functions. TCR-ESO and TCR 1G4-α95LY HHD T cells rejected established MC703.gCm tumors in HHDxRag1^-/-^ mice equally well (**Figure 3D**). The two TCR-transduced T cells expanded in the tumor-bearing mice, although TCR-ESO seemed to have higher expansion potency, and significantly more TCR-ESO T cells persisted in the blood of the HHDxRag1^-/-^ mice even after the tumor was rejected, compared to the TCR 1G4-α95LY T cells (**Figure 3E**).

In summary, TCR-ESO appeared to be comparable in NY-ESO-1 antigen recognition with the TCR 1G4-α95LY and more effective when compared to unmodified human-derived NY-ESO-1 TCRs. This observation supports the hypothesis that NY-ESO-1 reactive CD8 T cells are deleted by tolerance mechanisms and only low avidity T cells can be obtained from humans. TCR-ESO readily rejected large established murine tumor *in vivo* that was transduced with NY-ESO-1_157-165_ triple epitopes, comparable to that of TCR 1G4-α95LY.

### TCR-ESO T cells are specific to the NY-ESO-1_157-165_ peptide

TCR-ESO was derived from a T cell repertoire that was selected on murine self-antigens. Therefore, it was critical to analyze whether such TCRs cross-reacted to human self-antigens, which could lead to off-target toxicity. Different assays were adopted in order to assess the safety profile of TCR-ESO, i.e. Alanine scan, x-scan, LCL assay, A2 peptide library and primary human cell co-culture. Unmodified TCR 1G4 and the affinity-enhanced TCR 1G4-α95LY were included to analyze whether the two amino acid substitutes in the CDR3 region of the alpha chain of TCR 1G4-α95LY altered the specificity.

**Figure 4:**
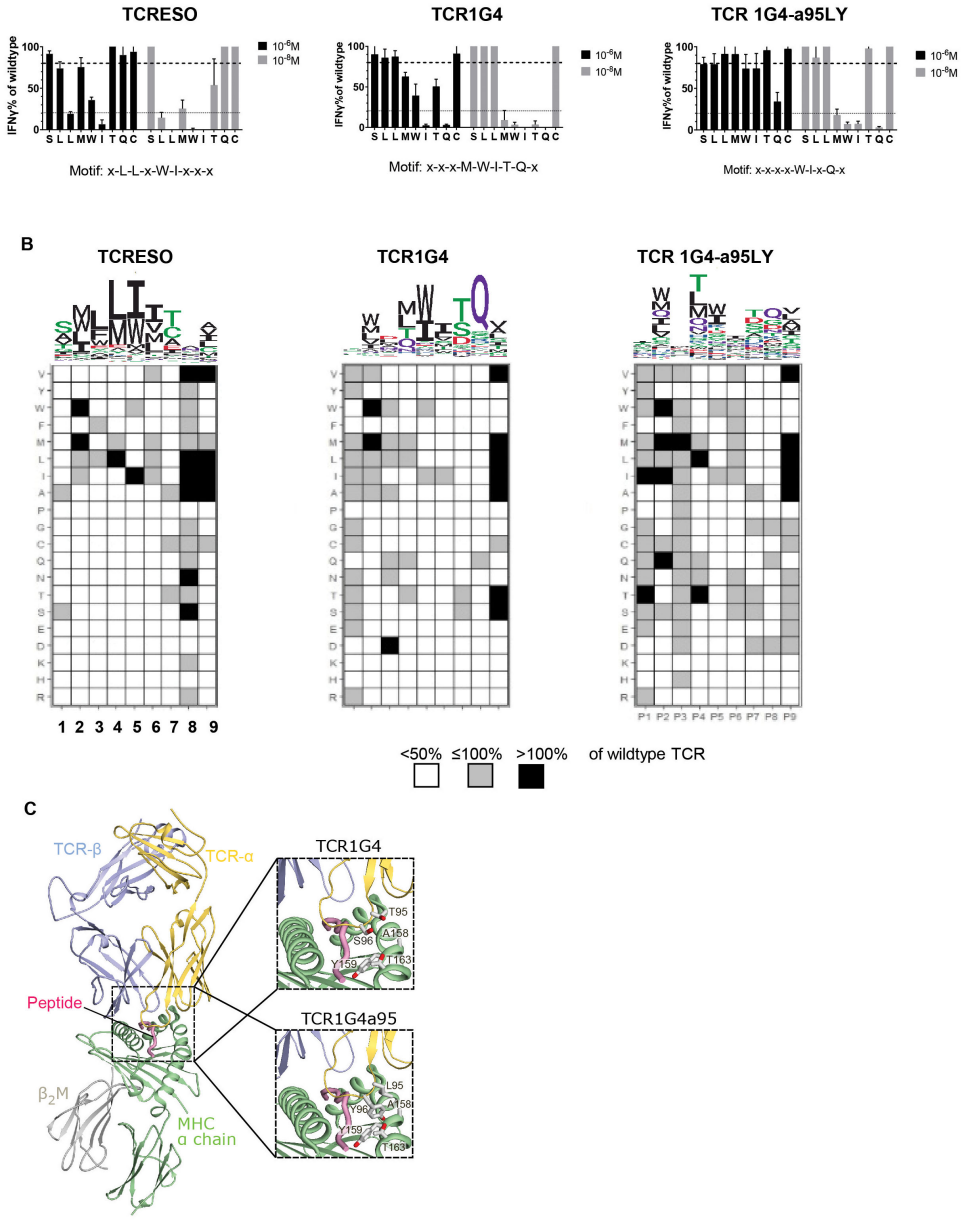
TCR cross-reactivity tests. **(A)** Alanine scan of human CD8 T cells transduced with TCR-ESO, TCR 1G4 and TCR 1G4-α95LY. Alanine scan peptides (see **Supplementary Table 1** for sequences) were loaded at indicated amount onto 10^4^ T2 cells and cultured with the same number of TCR-transduced human CD8 T cells overnight. IFNγ release was measured by ELISA. The IFNγ levels were normalized by T cell secreted IFNγ level stimulated with the wildtype peptide. The experiment was performed with three human donors, each in duplicates. A representative experiment is shown. **(B)** X-scan to determine the recognition motif of the NY-ESO specific TCRs. 10^4^ T2 cells were loaded with 10^-8^ M NY-ESO-1_157-165_ x-scan peptides, and co-cultured with 10^4^ TCR-ESO, TCR 1G4 or TCR 1G4a95 transduced huPBMCs overnight. Secreted IFNγ amounts were measured by ELISA. Upper: The recognition motif sequence Logo of the different TCRs. Lower: heatmaps indicating the changes of IFNγ level by the x-scan peptides compared to wildtype NY-ESO-1_157-165_. **(C)** Crystal structure of the TCR1G4-MHC class I complex (pdb 2bnq) (32) with a magnified view into the peptide binding site at the right. In the crystal structure, Thr95 and Ser96 in the TCR-α chain do not directly contact the MHC α-chain (see magnification at the right, top). The magnification at the right, bottom shows a model of the TCR1G4a95-MHC class I complex, in which Thr95 and S96 are exchanged to a preferred conformer of leucine and tyrosine, respectively. Compared to Thr95 and Ser96, Leu95 and Tyr96 have longer side-chains which may form a hydrophobic cluster with the opposite Ala158, Thr168 and Tyr159 of the MHC α-chain and therefore increase the affinity of the complex. Based on the model, the bound peptide is not expected to directly contact the mutated residues.

To study the recognition motif of TCR-ESO and derive the amino acids in NY-ESO-1_157-165_ which participate in the TCR interaction, we performed an alanine scanning study (**Supplementary Table 1**). A high (10^-6^ M) and low (10^-8^ M) concentration of the NY-ESO-1_157-165_ peptide, in which each amino acid was individually replaced with an alanine, was used to scan the recognition pattern of NY-ESO-1 TCR expressing human T cells. We considered a position to be a contact residue if the recognition at high concentration was lower than 80% and at low concentration lower than 20% of the recognition of the original peptide. With such criteria, TCR-ESO had a recognition motif of x-L-L-x-W-I-x-x-x, TCR 1G4 has a motif of x-x-x-M-W-I-T-Q-x and TCR 1G4-α95LY had a motif of x-x-x-x-W-I-x-Q-x (**Figure 4A**). Remarkably, TCR 1G4-α95LY completely lost dependency of the contact residue threonine at position 7 of the NY-ESO-1_157-165_ peptide compared to TCR 1G4. The contribution of other residues that are important for peptide recognition by TCR 1G4 (methionine, tryptophan, isoleucine and glutamine at position 4, 5, 6 and 8) also became less pronounced with TCR 1G4-α95LY. We searched for peptides which share the same TCR recognition motifs as the NY-ESO-1 TCRs from the human proteome using ScanProsite (37), and filtered the peptides based on their binding scores to HLA-A*02:01 according to NetMHC3.4 (38, 39). For TCR-ESO, all peptides that share the same sequences with murine peptides were excluded. 34 peptides that were potential HLA-A*02:01 binders were found for TCR-ESO, and 23 hits were found for TCR 1G4-α95LY. We did not find any peptides that share the same motif and were HLA-A2 binders for TCR 1G4 besides NY-ESO-1_157-165_ (all the gene names and peptide sequences are listed in **Supplementary Table 2**). We stimulated TCR-ESO and TCR 1G4-α95LY expressing human T cells with these related peptides. One peptide (SLLWISGA) from the immunoglobulin kappa variable 4-1 region (IGк4-1) evoked a TCR-ESO T cell response at a peptide concentration of 10^-8^ M, while TCR 1G4-α95LY T cells recognized peptides from IGк4-1, Opsin-3 and Troponin T (**Supplementary Figure 3A**).

To further investigate the binding characteristics of the NY-ESO-1 TCRs, we replaced each amino acid of NY-ESO-1_157-165_ by all other 19 amino acids, which generated 171 NY-ESO-1_157-165_ x-scan peptides. At position 1-7 of the epitope, only very few substituted amino acids evoked equivalent or superior responses in TCR-ESO T cells compared to the original NY-ESO-1_157-165_ antigen (**Figure 4B**). For example, TCR-ESO only recognized peptides that accommodated small nonpolar amino acids such as alanine or serine at position 1 (P1). At P3, P5 and P6, which were identified as contact positions for TCR-ESO recognition, only nonpolar amino acid substitutions were allowed, e.g., phenylalanine at P3. TCR 1G4 focused more on amino acids of P4-8, especially at P8, since only the glutamine from the original NY-ESO-1_157-165_ peptide evoked recognition by TCR 1G4. TCR 1G4-α95LY, on the other hand, tolerated quite a large number of amino acid substitutions at different positions of the peptide, except for P5 and P8 (**Figure 4B**).

To understand why TCR 1G4-α95LY exhibited such a promiscuous recognition, we modelled the TCR 1G4-α95LY-NY-ESO-1_157-165_/HLA-A0201 tertiary structure based on the published structure of the related TCR 1G4-NY-ESO-1_157-165_/HLA-A0201 (34). In the TCR 1G4 structure, Thr95 and Ser96 of the TCR-α do not contact the MHC α-chain. In TCR 1G4-α95LY, these two amino acids are replaced with a leucine and tyrosine, respectively. The structural model of the TCR 1G4-α95LY complex indicates that the exchanged Leu95 and Tyr96 can form additional hydrophobic interactions with Ala158, Thr168 and Trp159 of the HLA-A0201 molecule (**Figure 4C**), which is expected to increase the affinity of the TCR to the HLA-A0201 backbone.

We tested the three TCRs on an HLA-A0201 peptide library. These peptides were naturally processed and presented self-peptides identified by HPLC coupled MS from an HLA-A*02:01 positive cell line (40). None of the three TCRs showed cross-reactivity to any of the tested self-peptides (**Supplementary Figure 3B**).

To assess whether the TCRs cross-react to other HLA alleles, we co-cultured the T cells with a panel of lymphoblastoid cell lines (LCLs) that express different HLA-I molecules (HLA-I genotypes of the lines: **Supplementary Table 3**) (**Supplementary Figure 3C**). TCR-ESO and TCR 1G4 expressing human T cells did not recognize any of the LCL lines tested. In contrast, TCR 1G4-α95LY T cells were activated by two LCL lines, namely, TAB089 (HLA-A*02:07, HLA-B*46:01, HLA-C*01:02) and XC-IND (HLA-A*02:10, HLA-A*30:01, HLA-B*13:02, HLA-B*40:06, HLA-C*06:02, HLA-C*08:01). We cloned the individual HLAs from TAB089 and XC-IND into the K562 line, which is deficient in endogenous HLA expression (**Supplementary Figure 3D**, data not shown for recognition of K562 expressing HLA-B and C alleles). HLA-A02:07 and HLA-A02:10 could both present the NY-ESO-1_157-165_ epitope and be recognized by the three TCR expressing T cells, as shown in **Supplementary Figure 3D**. TCR-ESO T cells recognized the NY-ESO-1_157-165_/HLA-A02:07 or HLA-A02:10 at lower levels compared to the other two T cell lines (**Supplementary Figure 3D**). But interestingly, we observed reactivity of TCR 1G4-α95LY to the two alleles even without the exogenously loaded NY-ESO-1_157-165_ peptide (**Supplementary Figure 3D**). We additionally verified that TAB089 an XC-IND did not express NY-ESO-1 (data not shown), suggesting that TCR 1G4-α95LY is potentially cross-reactive to some peptide other than NY-ESO-1_157-165_ on HLA-A02:07 and HLA-A02:10.

### Assessment of safety of ATT targeting NY-ESO-1

It is critical that the tumor antigen is not expressed in normal cells to prevent on-target off-tumor toxicity. To this end, we examined by RT-PCR using a human tissue cDNA library, whether NY-ESO-1 is expressed in normal tissues. The limit of detection was 1 in 10^4^ cells (**Supplementary Figure 4A**). NY-ESO-1 cDNA was detected in testis and to a lower extent in liver and placenta (**Supplementary Figure 4B**). Placenta and testis are immune privileged sites. However, the detection of NY-ESO-1 cDNA in the liver drew our attention. We co-cultured the three TCR expressing T cells with human primary cells, including human bronchial epithelial cells (HBEpC), human cardiac myocytes (HCM-C), normal human dermal fibroblasts (NHDF-α) and hepatocytes (all HLA-A2^+^) that were isolated from five different donors. No recognition of the cells from different tissues were detected in this assay, unless the cells were loaded with the NY-ESO peptide (**Supplementary Figure 4C**). This suggested that NY-ESO-1 is a safe target for T cell therapy despite the trace amount of its cDNA expression in the liver.

## Discussion

ATT targeting NY-ESO-1 holds promise due to its restricted expression in normal tissues and the wide expression in various cancer types, for example oesophageal cancer, melanoma, synovial sarcoma (41). The potential central tolerance against the self-antigen represents an obstacle to isolate high potency TCRs against NY-ESO-1 from humans (26). *In vitro* selection of *in vitro* affinity-enhanced variants of human-derived TCRs is one way to overcome the problem, but it increases the risk of introducing undesired cross-reactivity to the TCRs (19). Here, we took advantage of the fact that there is no homolog of NY-ESO-1 in mice and isolated NY-ESO-1 reactive TCRs from the AB*ab*-A2 transgenic mice with a humanized T cell recognition system (24).

AB*ab*-A2 mice express a diverse human TCR repertoire. Previously, we showed that high avidity CD8 T cell responses could be evoked against an HLA-A*02:01 restricted MAGE-A1 epitope and in preliminary experiments against NY-ESO-1 (24, 25, 31). In this study, we further demonstrated the feasibility of isolation of highly potent TCRs against NY-ESO-1_157-165_/HLA-A*02:01 from the mice, analysed the safety profile of one of the TCRs and compared this TCR to a series of human-derived TCRs. Immunization evoked robust CD8 T cell responses in the mice and allowed cloning of their TCRs. We compared the avidity of TCR-ESO, which showed the highest potency among the mouse TCRs, to the TCRs that were isolated from human donors. Accordingly, the mouse-derived human TCR had higher functional avidity compared to a number of human-derived TCRs. These data suggest that high-affinity TCRs against NY-ESO are deleted in humans but not in mice, compatible with the notion that many cancer-testis antigens, including NY-ESO, are expressed in human medullary thymic epithelial cells (26), which are believed to be responsible for deletion of high-affinity T cells, even if they are specific for antigens rarely expressed in the adult. To note, NY-ESO-specific TCRs have been isolated from another mouse line with human TCRs (42). But the study did not attempt to address the question of T cell central tolerance to NY-ESO-1. The *in vivo* tumor model employed by Moore et al. differed from our *in vivo* tumor model. In Moore et al., NSG mice with a xenograft tumor were treated with human T cells transduced to express a NY-ESO-specific TCR. In most cases, tumors were not rejected (3 out of five mice). In xenograft-NSG models, treatment is started very early, i.e. in the study by Moore et al. on the same day as cancer cell injection. We employed a syngeneic tumor model, in which the molecules involved in T cell recognition, MHC I, TCR and tumor antigen epitope were of human origin, whereas all cells including the TCR-transduced and transferred T cells were of mouse origin. In our experiments, all mice bearing large established tumors of up to 500 mm^3^ in size and grown for three weeks rejected the tumor. We think that xenograft-NSG tumor models are problematic, because the transferred human T cells are allogeneic to the human cancer cells and xenogenic to the immune-deficient host. Furthermore, there are an undefined number of species-specific molecules involved in tumor rejection, impairing the function of human T cells in mice. IFNγ for example is required for tumor rejection and needs to act on the tumor vasculature (43). IFNγ is species-specific, so that the human IFNγ secreted by the T cells cannot act on mouse tumor stroma cells. Because of the different models, it is difficult to compare the NY-ESO specific TCRs between those described by Moore et al. and those described here.

We compared the properties of TCR-ESO with the clinically applied TCR 1G4-α95LY. The CD8 T cells transduced with either of the two TCRs exhibited similar activities, i.e. they had similar peptide sensitivity and they recognized NY-ESO-1 expressing tumor cells with comparable efficacy. Both TCRs showed superior function when compared to TCR 1G4. Both TCR-ESO and TCR 1G4-α95LY expressing mouse T cells can reject large, established syngeneic tumors in HHDxRag^-/-^ mice. However, CD8 T cells expressing TCR-ESO expanded to and persisted *in vivo* in significantly higher T cell numbers compared to TCR 1G4-α95LY even 35 days after T cell transfer. From previous experiments, we knew that rejection of large established tumors by adoptively transferred T cells required responsiveness of tumor endothelial cells but not other tumor stroma or cancer cells to IFNγ and that destruction of the tumor vasculature was the first measurable event in the tumor microenvironment, before macroscopic tumor regression was observed (43). It seems likely that tumor rejection by the TCR-ESO involved a similar mechanism. Another important factor to be considered while selecting TCRs for ATT is safety. TCRs that are cross-reactive to other antigens can cause off-target toxicity in the T cell recipients (18, 19, 44). This is especially important, since TCR-ESO is not negatively selected against human antigens. Due to the protein sequence discrepancy between human and mice (45), there might be potential cross-reactivity to human self-antigens that are related to NY-ESO-1. Therefore, we employed a series of experiments with the aim to minimize the risk of off-target toxicity. These experiments included alanine scan and x-scan to determine the TCR contact residues of the NY-ESO peptide, searching the human proteome for peptides with the same TCR contact motif and testing these peptides for recognition by NY-ESO specific TCRs. Furthermore, the TCRs were tested for recognition of a library of self-peptides and, for testing HLA alloreactivity, a library of human LCL cell lines covering a large number of MHC I and MHC II alleles. TCR-ESO and TCR 1G4 showed typical TCR recognition motifs, although the motifs were quite different from each other. On the other hand, TCR 1G4-α95LY revealed a rather degenerated pattern of antigen recognition, which could be seen in both alanine scan and x-scan. This is a similar approach as in a previous report studying TCR 1G4-α95LY recognition fingerprints (46), although we synthesized x-scan peptides with the original cysteine in position 9, as opposed to the valine version in the previous study. The general increase in reactivity to many peptides in the x-scan of TCR 1G4-α95LY can best be explained by the two amino acid substitutions in the CDR3 of the TCRα chain primarily increasing the affinity to the HLA-A*02:01 molecule and not the NY-ESO peptide. Structure prediction as well as cross-reactivity to HLA-A*02:07 and HLA-A*02:10 confirmed this assumption (47). Since the function of T cells *in vivo* is influenced by tonic signalling by self-MHC/self-peptide, the increased affinity of TCR 1G4-α95LY for HLA-A*02:01 could affect function and persistence of the T cells in clinical trials. In brief, based on these cumulative assays, the mouse-derived TCR-ESO had a good safety profile, whereas the human affinity-matured TCR 1G4-α95LY appeared to have increased affinity to HLA-A2, leading to general increased affinity in the alanine scan and x-scan as well cross-reactivity to some HLA-I alleles.

Currently, two more affinity-enhanced NY-ESO-1 TCRs besides the TCR 1G4-α95LY(c259) are in clinical trials (48, 49), both reported no treatment-related serious adverse events. The overall response rates reported seem to be similar, if not worse than with TCR 1G4-α95LY, which showed similar TCR potency with TCR-ESO both *in vitro* and *in vivo*. As mentioned earlier, TCR affinity maturation increased the risks of TCR off-target toxicity and even lead the T cells into tolerance-like state and dampen the potencies of the engineered T cells (18, 19, 50). TCR-ESO, on the contrary, was selected not based on the affinity, but rather on the functionality of the T cells. In addition, TCR-ESO comes from a TCR repertoire that has been negatively selected against TCR clones that have too high affinity against HLA-A*0201 molecule, further excludes the possibility of tolerance without even the presence of the antigen (50).

In summary, we isolated HLA-A*02:01 restricted NY-ESO-1_157-165_ specific TCRs from non-tolerant mice with a humanized T cell recognition system and a diverse human TCR repertoire. One of the TCRs, TCR-ESO, exhibits a superior efficacy and safety profile. By comparing TCR-ESO to a clinically relevant TCR, TCR 1G4-α95LY, we demonstrate the feasibility and advantages of isolating TCRs from the humanized transgenic mice.

## Data Availability

The original contributions presented in the study are included in the article/**Supplementary Material**. Further inquiries can be directed to the corresponding author.
