# Supplementary material for: Generation of effective and specific human TCRs against tumor/testis antigen NY-ESO-1 in mice with humanized T cell recognition system
Source: Front Immunol. 2024 Dec 24;15:1524629. doi: 10.3389/fimmu.2024.1524629 (PMC11703889; doi:10.3389/fimmu.2024.1524629)
Supplement: Supplementary file 1 [file DataSheet1.docx]

Supplementary Material

## Supplementary Figures


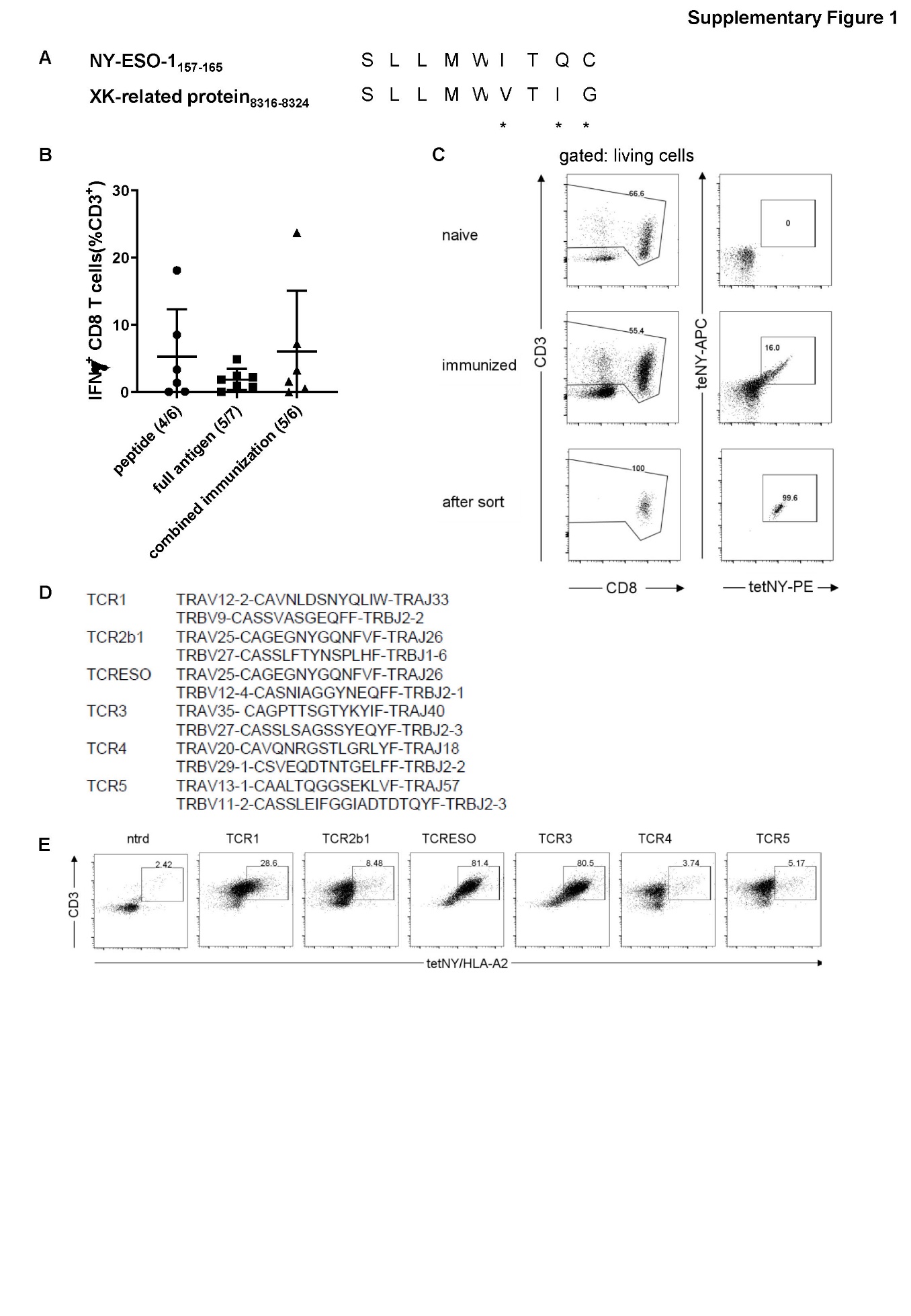


**Supplementary** **Figure 1. (A)** Sequence comparison of NY-ESO-1_156-165_ and the murine peptide XK-related protein. Different amino acids are marked by stars (*). **(B)** Comparison of CD8 T cell responses of AB*ab*-A2 mice treated with different immunization regimens (peptide, full NY-ESO-1 cDNA gene gun and combined immunization). Mice were immunized and boosted once with an interval of at least 4 weeks. Blood was taken, cells re-stimulated *ex vivo* for 14 hours by NY-ESO-1_157-165_ peptide and stained for IFNγ production 7 days after the boost. Number of responding mice per group are indicated in bracket. **(C)** FACs sorting strategy for identification of NY-ESO-1_157-165_ specific CD8 T cells and the purity of the sorted T cells. AB*ab*-A2 mice were twice immunized with NY-ESO-1 full length antigen and boosted once with anchor-modified NY-ESO-1_157-165_ peptide. Seven days after the boost, splenocytes were isolated and stained for mouse CD3, CD8 and NY-ESO-1_157-165_/HLA-A2K^b^ tetramer-APC and –PE. The splenocytes from a naïve mouse was used as negative control. Gate: left panel: living cells; right panel: CD3^+^ cells. **(D)** Paired TCR sequences from the sorted T cells of NY-ESO-1 immunized AB*ab*-A2 mice identified by TCR capture assay. **(E)** NY-ESO-1_157-165_/HLA-A2 tetramer staining of TCR transduced Jurkat-CD8 cells. Jurkat-CD8 cells were transduced with different TCRs from (D), and stained for CD3 and NY-ESO-1_157-165_ tetramer. Jurkat-CD8 cells without transduced TCR (ntrd) were used as negative control.


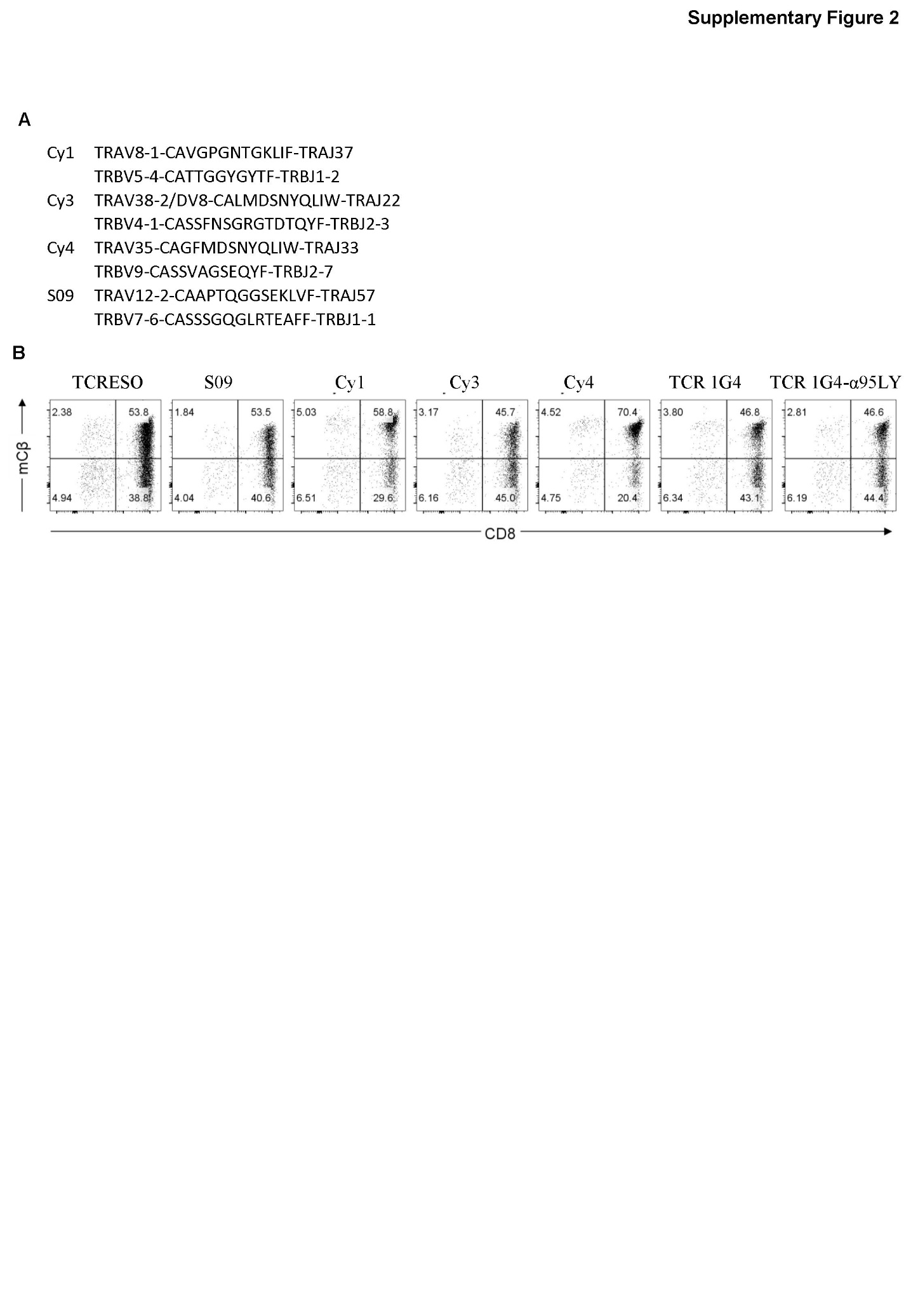


**Supplementary Figure 2. (A)** Paired TCR sequences from NY-ESO-1_157-165_ TCRs of human source. **(B)** FACs staining of TCR transduced human PBMCs with murine TCRβ constant region antibody. Gate: 7-AAD^-^CD3^+^ lymphocytes. The transduction and staining were performed on three human donors. One representative plot was shown for each TCR on CD8 T cells.


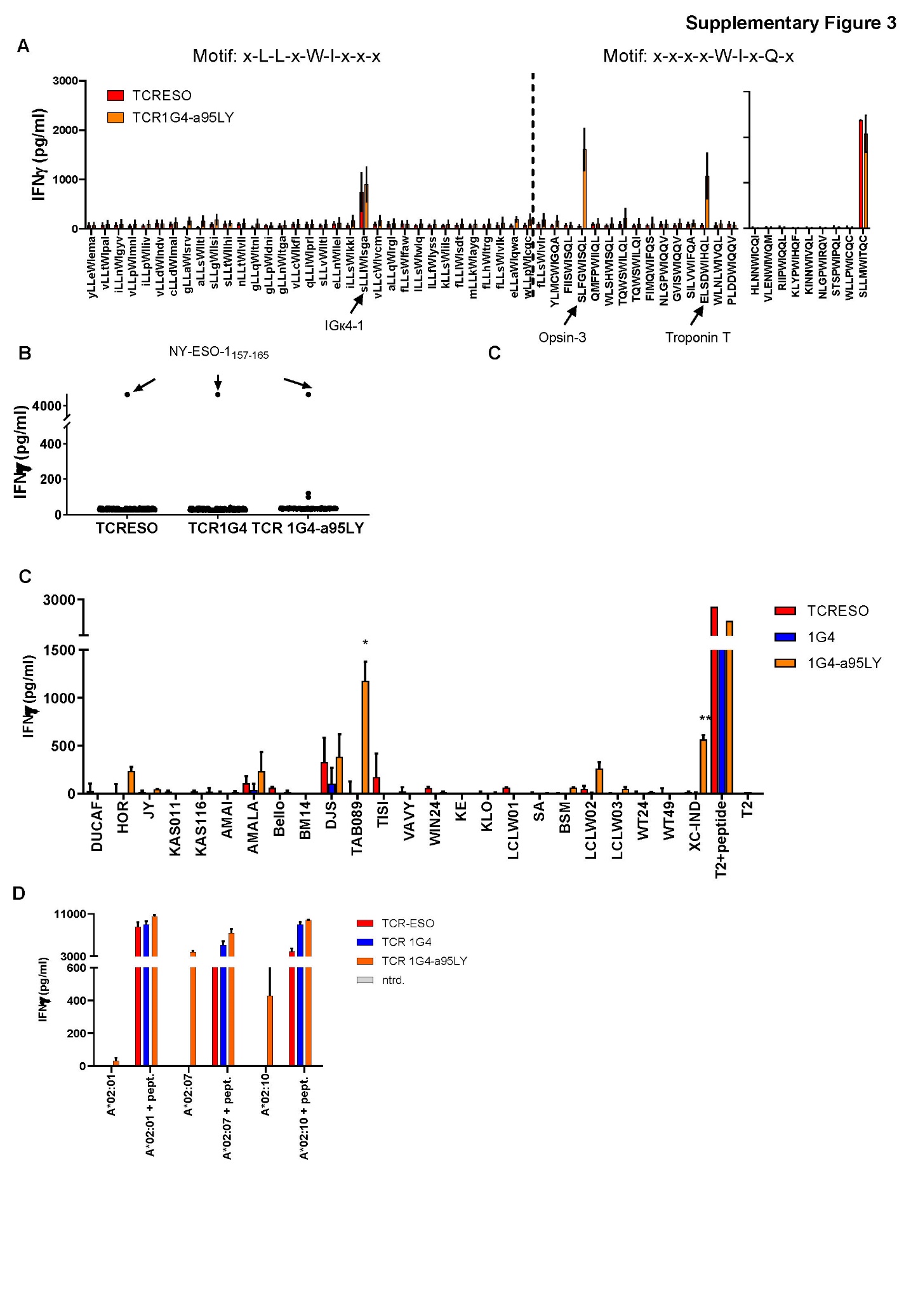


**Supplementary Figure 3. (A)** Cross-reactivity test of peptides that share the recognition motifs with TCRESO and TCR1G4a95. 10^4^ TCRESO and TCR 1G4-α95LY transduced huPBMCs were co-cultured with T2 cells loaded with the peptide analogues overnight. IFNγ levels were measured by ELISA. The experiment was repeated with PBMCs from two human donors. (C) Recognition of K562 cells transduced with a single HLA allele with TCR transduced human PBMCs as in Figure 4. 5x10^4^ K562 cells transduced with indicated HLA alleles with or without loading of NY-ESO-1_157-165_ peptide were cultured with 5x10^4^ whole human PMBCs transduced with TCR-ESO, TCR1G4 or TCR 1G4-α95LY overnight. IFNγ level was then measure by ELISA. The experiment was performed with three human donors with duplicate for each donor. Data from one representative donor was shown. **(B)** T cell recognition of peptide library. The peptide library composed of 9-mer peptides that are strong HLA-A*02:01 binders and were identified by HLA immunoprecipitation from cell lines. 10^-6^M of each peptide from the library was loaded onto 10^4^ T2 cells, cultured with 5x10^4^ whole human PMBCs transduced with TCR-ESO, TCR 1G4 or TCR 1G4-α95LY overnight. IFNγ release was then measured by ELISA. The experiment was performed with three human donors with duplicate for each donor. **(C)** Allo-reactivity test. 10^4^ human PBMCs transduced with TCR-ESO, TCR 1G4 and TCR 1G4-α95LY were cultured with 10^4^ LCL lines expressing different HLA alleles overnight. IFNγ amounts were then measured by ELISA. Non-transduced human PBMCs were used to determine the baseline recognition of LCLs and was subtracted from the measurement. The experiment was performed with five human donors with duplicates for each donor. Data from one representative donor is shown. **(D)** Recognition of K562 cells transduced with a single HLA allele by TCR transduced human PBMCs. 5x10^4^ K562 cells transduced with indicated HLA genes with or without loading of NY-ESO-1_157-165_ peptide were cultured with 5x10^4^ human PMBCs transduced with TCR-ESO, TCR 1G4 or TCR 1G4-α95LY overnight. IFNγ amounts were then measured by ELISA. The experiment was performed with three human donors in duplicates for each donor. Data from one representative donor was shown.


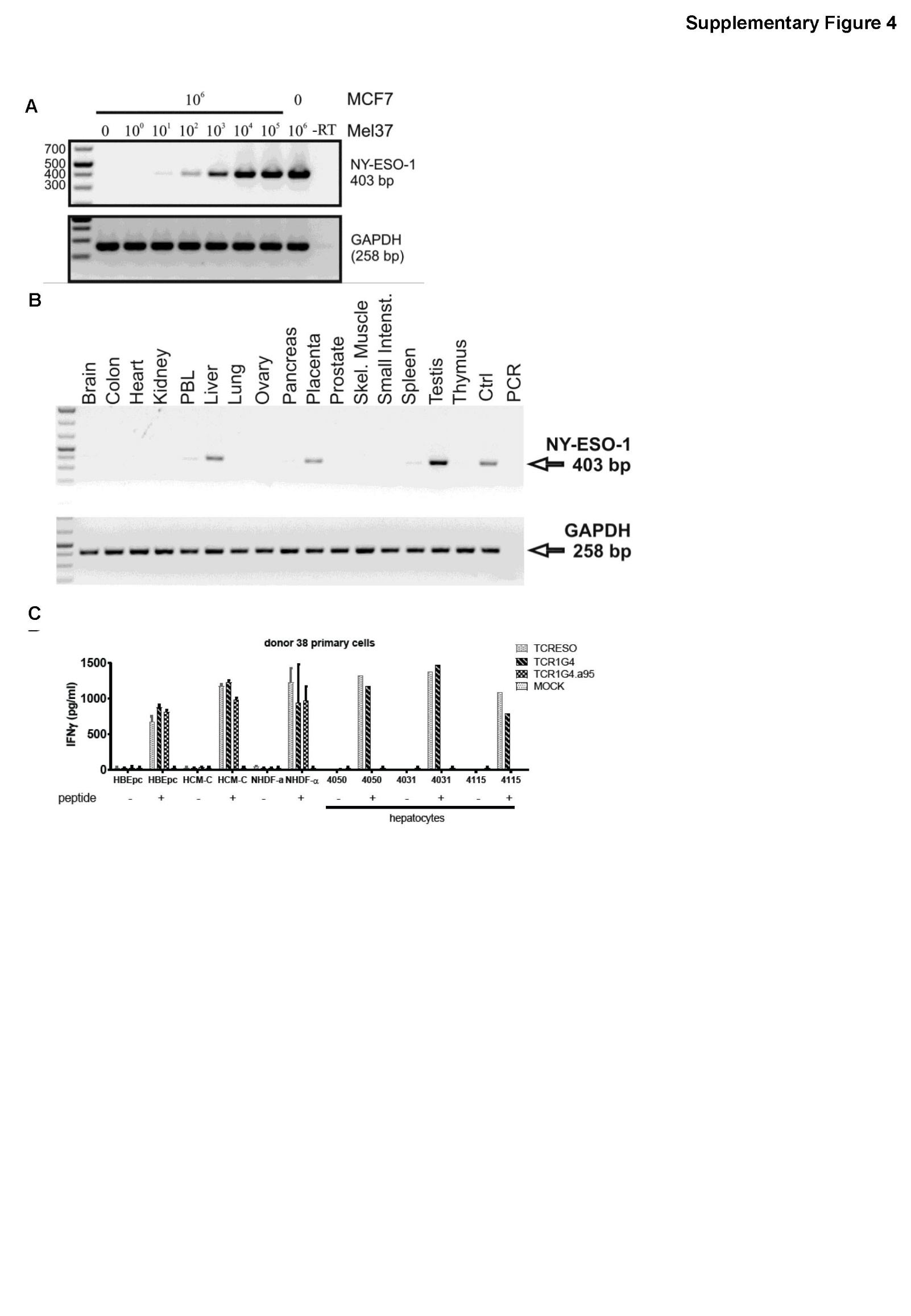


**Supplementary Figure 4. (A)** Sensitivity test of the rt-PCR for detection of NY-ESO-1. **(B)** NY-ESO-1 and LAGE-1 cDNA expression in different tissues from human tissue cDNA pool. **(C)** T cell recognition of human primary cells from different origins of healthy human donors. Three human donors were transduced with NY-ESO-1_157-165_ TCRs, 10^4^ whole transduced huPMBCs were cultured with 5x10^3^ primary human cells overnight. Human IFNγ ELISA was performed with the supernatant. One representative experiment was shown. HBEpc: human bronchial epithelial cells; HCM-C: human cardiomyocytes; NHDF: normal human dermal fibroblasts.

**Supplementary table 1. Peptide sequences used for alanine scan**

| S | L | L | M | W | T | I | Q | C |
| --- | --- | --- | --- | --- | --- | --- | --- | --- |
| A | L | L | M | W | T | I | Q | C |
| S | A | L | M | W | T | I | Q | C |
| S | L | A | M | W | T | I | Q | C |
| S | L | L | A | W | T | I | Q | C |
| S | L | L | M | A | T | I | Q | C |
| S | L | L | M | W | A | I | Q | C |
| S | L | L | M | W | T | A | Q | C |
| S | L | L | M | W | T | I | A | C |
| S | L | L | M | W | T | I | Q | A |

**Supplementary table 2.**

**(a) Peptides that shared TCRESO recognition motif in human and not in mouse, and have HLA-A*0201 binding IC50<500nM**

**x-L-L-x-W-I-x-x-x**

| protein | start | end | sequence |
| --- | --- | --- | --- |
| sp\|P07550\|ADRB2_HUMAN | 309 | 317 | ilLnWIgyv |
| sp\|Q4KMQ2\|ANO6_HUMAN | 624 | 632 | vlLpWImnl |
| sp\|Q9NPZ5\|B3GA2_HUMAN | 11 | 19 | ilLpWIliv |
| sp\|Q9Y426\|C2CD2_HUMAN | 62 | 70 | alLsWIltl |
| sp\|Q8TAB5\|CA216_HUMAN | 179 | 187 | alLqWIrgl |
| sp\|Q9H5L9\|CE066_HUMAN | 117 | 125 | glLqWItnl |
| sp\|Q8TDX6\|CGAT1_HUMAN | 7 | 15 | glLaWIsrv |
| sp\|A6NM45\|CLD24_HUMAN | 17 | 25 | slLgWIlsi |
| sp\|Q8N8G6\|CO054_HUMAN | 38 | 46 | klLsWIils |
| sp\|Q9ULK0\|GRID1_HUMAN | 7 | 15 | wlLpWIcqc |
| sp\|Q15751\|HERC1_HUMAN | 263 | 271 | ylLeWIema |
| sp\|Q9NPH9\|IL26_HUMAN | 158 | 166 | ilLsWIkkl |
| sp\|Q96J84\|KIRR1_HUMAN | 3 | 11 | slLvWIltl |
| sp\|P06312\|KV401_HUMAN | 10 | 18 | slLlWIsga |
| sp\|P06315\|KV502_HUMAN | 10 | 18 | flLlWIsdt |
| sp\|Q9UPN3\|MACF1_HUMAN | 6889 | 6897 | elLaWIqwa |
| sp\|O60287\|NPA1P_HUMAN | 1853 | 1861 | slLtWIlhi |
| sp\|Q9UHC9\|NPCL1_HUMAN | 839 | 847 | flLhWItrg |
| sp\|Q96SN7\|ORAI2_HUMAN | 166 | 174 | vlLcWIkfl |
| sp\|Q9HBI1\|PARVB_HUMAN | 92 | 100 | vlLdWIndv |
| sp\|Q75T13\|PGAP1_HUMAN | 821 | 829 | nlLtWIvll |
| sp\|Q92521\|PIGB_HUMAN | 137 | 145 | qlLiWIprl |
| sp\|Q6GYQ0\|RGPA1_HUMAN | 1394 | 1402 | clLdWImal |
| sp\|Q6PJF5\|RHDF2_HUMAN | 763 | 771 | glLpWIdni |
| sp\|Q86VV8\|RTTN_HUMAN | 1164 | 1172 | elLnWIlel |
| sp\|Q9BZW2\|S13A1_HUMAN | 296 | 304 | llLsWIwlq |
| sp\|A1A5C7\|S22AN_HUMAN | 100 | 108 | vlLtWIpal |
| sp\|Q8IY50\|S35F3_HUMAN | 192 | 200 | flLsWIvlr |
| sp\|A4IF30\|S35F4_HUMAN | 290 | 298 | flLsWIvlk |
| sp\|Q15858\|SCN9A_HUMAN | 1241 | 1249 | mlLkWIayg |
| sp\|Q6ZQN7\|SO4C1_HUMAN | 318 | 326 | flLsWIfaw |
| sp\|Q7Z7N9\|T179B_HUMAN | 80 | 88 | llLfWIyss |
| sp\|Q7Z5H5\|VN1R4_HUMAN | 131 | 139 | vlLcWIvcm |
| sp\|Q5TIE3\|VW5B1_HUMAN | 3 | 11 | glLnWItga |

**(b) Peptides that shared TCR1G4a95 recognition motif and have HLA-A*0201 binding IC50<500nM**

| protein | start | end | sequence |
| --- | --- | --- | --- |
| sp\|Q8N4J0\|CARME_HUMAN | 252 | 260 | klypWIhQf |
| sp\|O75638-2\|CTAG2_HUMAN | 157 | 165 | sllmWItQc |
| sp\|O14578\|CTRO_HUMAN | 152 | 160 | stspWIpQl |
| sp\|Q7L576-2\|CYFP1_HUMAN | 120 | 128 | qmfpWIiQl |
| sp\|P15924\|DESP_HUMAN | 356 | 364 | tqwsWIlQi |
| sp\|Q03001\|DYST_HUMAN | 785 | 793 | tqwsWIlQl |
| sp\|Q03001\|DYST_HUMAN | 1301 | 1309 | plddWIqQv |
| sp\|P15328\|FOLR1_HUMAN | 112 | 120 | nlgpWIqQv |
| sp\|P41439\|FOLR3_HUMAN | 112 | 120 | nlgpWIrQv |
| sp\|Q9ULK0\|GRID1_HUMAN | 7 | 15 | wllpWIcQc |
| sp\|Q7Z2Y8\|GVIN1_HUMAN | 613 | 621 | fimqWIfQs |
| sp\|P11150\|LIPC_HUMAN | 95 | 103 | vlenWIwQm |
| sp\|O60244\|MED14_HUMAN | 488 | 496 | riipWIqQl |
| sp\|P22897\|MRC1_HUMAN | 771 | 779 | hlnnWIcQi |
| sp\|Q5T3U5\|MRP7_HUMAN | 905 | 913 | wlshWIsQl |
| sp\|Q9H1Y3-2\|OPN3_HUMAN | 122 | 130 | slfgWIsQl |
| sp\|Q4G0N8\|SL9C1_HUMAN | 628 | 636 | fiisWIsQl |
| sp\|O95473\|SNG4_HUMAN | 153 | 161 | silvWIfQa |
| sp\|Q9H330\|TM245_HUMAN | 369 | 377 | wlnlWIvQl |
| sp\|Q8IU80\|TMPS6_HUMAN | 801 | 809 | gvisWIqQv |
| sp\|P13805\|TNNT1_HUMAN | 225 | 233 | elsdWIhQl |
| sp\|Q6PGP7\|TTC37_HUMAN | 894 | 902 | ylmcWIgQa |
| sp\|P22415\|USF1_HUMAN | 214 | 222 | kinnWIvQl |

**Supplementary table 3. The HLA genotypes of the LCL lines**

| **B-LCL** | **A*** | | **B*** | | **Cw*** | |
| --- | --- | --- | --- | --- | --- | --- |
| **SPO** | 02:01 |  | 44:02 |  | 05:01 |  |
| **KAS011** | 01:0101 |  | 37:01 |  | 06:02 |  |
| **BM14** | 03:01 |  | 07:02 |  | 07:02 |  |
| **AMALA** | 02:1701 |  | 15:010101 |  | 03:03 |  |
| **MT14B** | 31:01:00 |  | 40:01 |  | 03:04 |  |
| **SA** | 24:020101 |  | 07:0201 |  | 07:02 |  |
| **HOR** | 33:0301 |  | 44:0301 |  | 14:03 |  |
| **BSM** | 02:0101 |  | 15:010101 |  | 03:0401 |  |
| **KE** | 02:01 | 29:02 | 44:03 | 44:05 | 02:02 | 16:01 |
| **TISI** | 24:020101 |  | 35:08 |  | 04:01 |  |
| **WIN** | 01:01 |  | 57:0101 |  | 06:02 |  |
| **KAS116** | 24:020101 |  | 51:01 |  | 12:03 |  |
| **TAB089** | 02:07 |  | 46:01 |  | 01:02 |  |
| **XC-IND** | 02:10 | 30:01 | 13:02 | 40:06 | 08:01 | 06:02 |
| **DUCAF** | 30:02 |  | 18:01 |  | 05:01 |  |
| **WT24** | 02:0101 |  | 27:0502 |  | 02:0202 |  |
| **AMAI** | 68:02 |  | 53:01 |  | 04:01 |  |
| **DJS** | 02:01 | 03:01 | 35:01 | 37:02 | 04:01 | 06:02 |
| **VAVY** | 01:01 |  | 08:01 |  | 07:01 |  |
| **RML** | 02:04 |  | 51:0101 |  | 15:02 |  |
